# An autonomous compartmental model for accelerating epidemics

**DOI:** 10.1371/journal.pone.0269975

**Published:** 2022-07-18

**Authors:** Nazmi Burak Budanur, Björn Hof

**Affiliations:** 1 Max Planck Institute for the Physics of Complex Systems (MPIPKS), Dresden, Germany; 2 Institute of Science and Technology Austria (IST Austria), Klosterneuburg, Austria; Montclair State University, UNITED STATES

## Abstract

In Fall 2020, several European countries reported rapid increases in COVID-19 cases along with growing estimates of the effective reproduction rates. Such an acceleration in epidemic spread is usually attributed to time-dependent effects, e.g. human travel, seasonal behavioral changes, mutations of the pathogen etc. In this case however the acceleration occurred when counter measures such as testing and contact tracing exceeded their capacity limit. Considering Austria as an example, here we show that this dynamics can be captured by a time-independent, i.e. *autonomous*, compartmental model that incorporates these capacity limits. In this model, the epidemic acceleration coincides with the exhaustion of mitigation efforts, resulting in an increasing fraction of undetected cases that drive the effective reproduction rate progressively higher. We demonstrate that standard models which does not include this effect necessarily result in a systematic underestimation of the effective reproduction rate.

## Introduction

Severe acute respiratory syndrome coronavirus 2 (SARS-CoV-2) [1] and its variants [2] continue to challenge our lives [3] even after the rollout of several effective vaccines [4]. As of Fall 2021, non-pharmaceutical interventions such as mask mandates and travel restrictions at varying degrees still remain in place around the globe [3]. These interventions are guided by almost-real-time assessment of the ongoing epidemiological situation which rely on surveillance data and mathematical models and, are thus, prone to their uncertainties and shortcomings [5]. It is, therefore, crucial for decision making that the epidemiological models are sufficiently *simple* to be used in a fast-changing environment while containing the necessary amount of *complexity* to capture all essential features of the real epidemic.

Simple epidemic models divide a population into “compartments” according to individuals’ epidemiological status and specify the rules by which the disease progresses within an individual and spreads over the population [6]. In the most basic form, these rules are given as transition rates between the compartments which can be translated into a set of ordinary differential equations (ODEs). One such model is the SEIR model where the compartments correspond to those who are susceptible (S) to infection, exposed (E) to the pathogen (but not yet a spreader), infectious (I), and removed (R) from epidemic dynamics (dead or immune). The SEIR model can capture the initial exponential increase of infections when the majority of a population is susceptible and the subsequent slow down of spreading due to the continuously increasing removed population.

In Fall 2020, the second wave of the COVID-19 in Austria exhibited a remarkably different behavior than the one that we described above. What appeared to be slow-but-steady initial increase was followed by an *acceleration* of the epidemic indicated by a faster-than-exponential growth in case numbers [7]. For simple mathematical models, such an observation indicates a change in rules, i.e. increased transmissibility, which can be due to seasonality [8, 9] or occurrence of more infectious variants [10]. In contrast to such expectations, here we show that the accelerating increase of COVID-19 cases reported in Austria during Fall 2020 can be captured in an autonomous compartmental model described by a time-independent (deterministic) set of ODEs. As we explain in the following, the key modeling ingredient for accelerating epidemics is explicit inclusion of mitigation efforts and their capacity limitations within the model dynamics.

Since the early stages of the pandemic, case and contact isolation has been one of the primary public health responses [11]. Using stochastic agent-based epidemic models on networks, Scarselli et al. [12] showed that while testing and contact isolation slow down an epidemic, limiting the number of available tests fundamentally alter its nature by changing the epidemic transition for large populations (in the thermodynamic limit) from gradual (second order) to sudden (first order). The driving mechanism of this qualitative change was the acceleration of transmissions when the models’ testing capacity were exhausted. In the present work, we introduce the *SEIRTC model* which, in addition to the *S*, *E*, *I*, and *R*, includes separate compartments for the tested individuals (T) and confirmed cases (C) similar to [12]. Differently from the stochastic network models used in [12], the dynamics of the SEIRTC model is determined by a set of ODEs, rendering it suitable for working with real data. In other words, our aim in the present paper is to present a simple way of incorporating capacity-limited interventions to the standard epidemic models so that it can be utilized to explain the real-world observations without resorting to complex network-based models. In this sense, our approach is similar to that of Arino et al. [13], who presented an extension of the SEIR model that includes treatment as a counter-acting measure to explain influenza data. We show that the SEIRTC model can be fitted to the COVID-19 surveillance data published by the Austrian Agency for Health and Food Safety (AGES) [14] and capture the epidemic acceleration observed in Fall 2020 without the need for a temporal modification of the infectiousness. Our results suggests that during this period, the effective reproduction rate, i.e. the average number of secondary cases originating from a primary one after the initial uncontrolled spread period, was systematically underestimated.

## Methods

**Fig 1:**
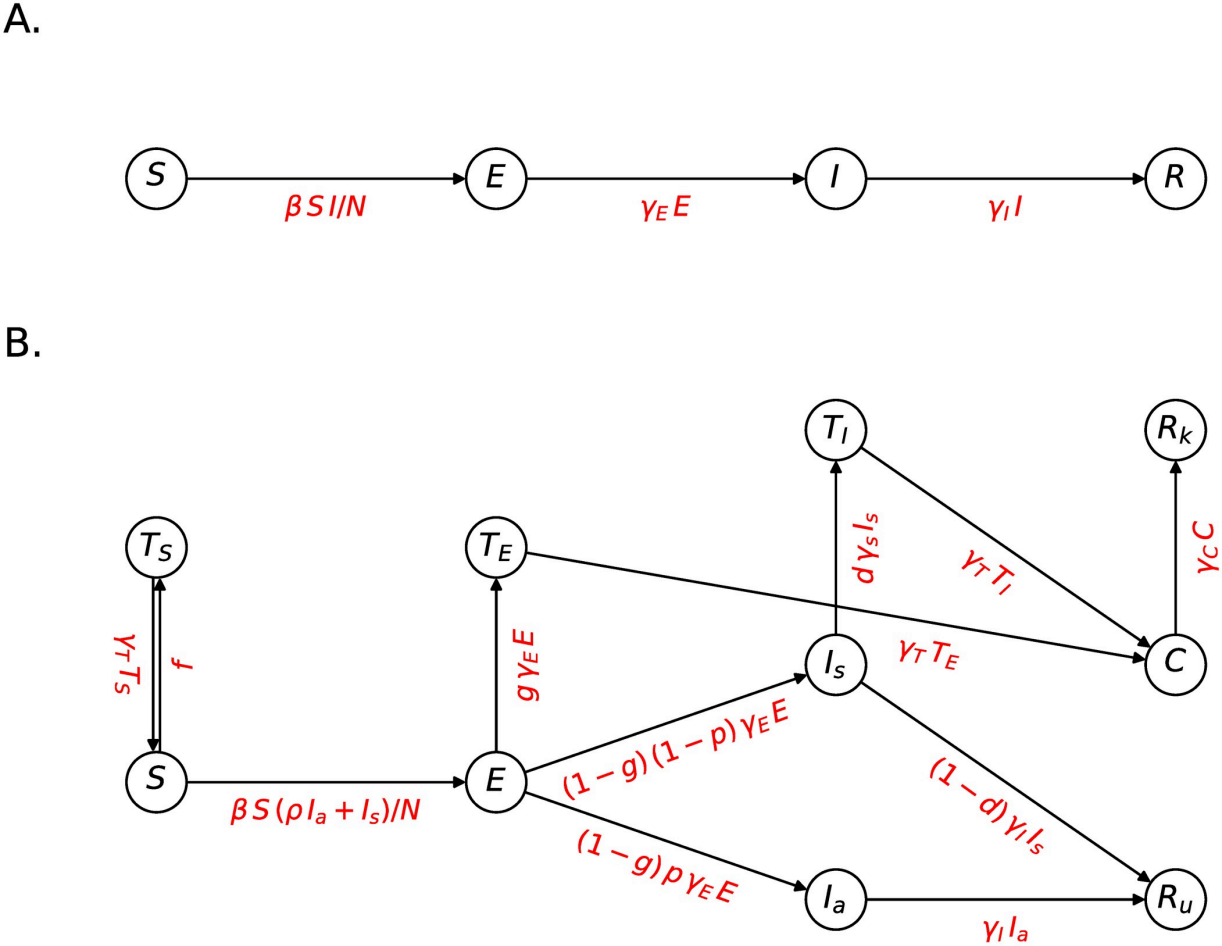
State transition diagrams of SEIR (A) and SEIRTC (B) models where the encircled letters denote the compartments into which the population is divided and the arrows along with their labels underneath indicate the transition rates between the compartments. The compartments are *S*: susceptible, *E*: exposed, *I*: infectious, *R*: removed (recovered or dead), *I*_*a*/*s*_: a/symptomatic infectious, *R*_*u*/*k*_: un/known removed, *T*_*S*/*E*/*I*_: tested susceptible/exposed/infectious, *C*: case.

We begin with a brief recapitulation of the standard SEIR model which forms the basis of our SEIRTC model to follow. For a detailed treatment, we refer the reader to [6, 15]. The SEIR model is represented by the state transition diagram in Fig 1A and the corresponding ODEs can be written by expressing the rates of changes in compartments’ populations according to the transition rates implied by the annotated arrows as

$$\begin{array}{c} \dot{S}=-\beta IS/N,\;\dot{E}=\beta IS/N-\gamma_{E}E,\;\dot{I}=\gamma_{E}E-\gamma_{I}I,\;\dot{R}=\gamma_{I}I, \end{array}$$ (1)

where, ˙ denotes derivative with respect to time, *N* = *S* + *E* + *I* + *R* is the population, *β* is the transmission parameter, and *γ*_*E*_ and *γ*_*I*_ are the inverse latent and infectious times, respectively. The transmission parameter *β* can be interpreted as the number of interactions per person per unit time multiplied by the transmission probability at each interaction [6]. The underlying assumption of SEIR model is that the population is well mixed, thus, the effects solely due to the network heterogeneities are neglected.

We obtain the SEIRTC model shown in Fig 1B by the following series of modifications to the SEIR. First, we split the infectious individuals *I* into two sub-compratments, namely *I*_*s*_ “symptomatic” and *I*_*a*_ “asymptomatic”, the latter of whom are those who show no symptoms throughout their infectious period and spread the disease at a relative risk *ρ* as implied by the *S* → *E* term. At this stage, our model is equivalent to the SLIAR model of Arino et al. [13]. Next, we incorporate testing into our model by introducing the compartments *T*_*S*_, *T*_*E*_, and *T*_*I*_, where the subscripts refer to the epidemiological state of the individuals who are tested. We assume that testing with symptoms also invokes isolation, i.e. the individuals in the *T*_*I*_ state can no longer spread the disease. The susceptibles who are tested (*T*_*S*_) return to *S*, whereas the exposed and infectious individuals become “cases” (*C*) after being tested. Finally, the removed individuals are split into “known” (*R*_*k*_) and “unknown” (*R*_*u*_) parts for convenience in presenting our results to follow.

Similar to those in the SEIR model, the parameters *γ*_*x*_ of the SEIRTC model refer to the inverse mean lifetime at the compartment *x*, with the exception *γ*_*s*_ which is the inverse mean symptom onset time, at which point a symptomatic infectious individual becomes detectable. Hence, this delay term accounts for presymptomatic transmissions which are believed to play a significant role in spreading COVID-19 [16]. *γ*_*T*_ is the test turn-over time, i.e. the time from the administration of a test to its outcome. Finally, *γ*_*C*_ is not an independent parameter but is determined as

$$\begin{array}{c} \gamma_{C}={\left[\gamma_{I}^{-1}-\frac{T_{E}\gamma_{T}^{-1}+T_{I}\left(\gamma_{s}^{-1}+\gamma_{T}^{-1}\right)}{T_{E}+T_{I}}\right]}^{-1}, \end{array}$$ (2)

where we assume that at time *t*, the ratio of cases that are identified before and after developing symptoms are proportional to the number of individuals in *T*_*E*_ and *T*_*I*_, respectively. In doing so, we neglect a delay by $\gamma_{T}^{-1}$ and avoid working with delay-differential equations which are complicated to work with numerically.

The transition rates in Fig 1B have several probabilistic factors. These are *p*: probability of an asymptomatic infection, *ρ*: relative risk of transmission from an asymptomatic individual, *d*: probability of detecting a symptomatic infectious individual via testing, *g*: probability of detecting an exposed individual via contact tracing before becoming infectious. All but the last one of these probabilities are independent parameters to be determined via literature estimates or model fitting. Because the detection of an infection before developing any symptoms can only be possible via contact tracing, the probability *g* is a complex function of the number of (un)identified infections, the underlying social network structure, and the contact tracing policy and capacity. Since these details are not within the scope of the present model, here, we resort to an ansatz that is based on two simplifying assumptions: (i) The probability of detecting a case is proportional to the ratio *C*/(*C* + *I*_*s*_ + *I*_*a*_) of known cases to those that are undetected at a given time, (ii) Total contact tracing capacity is limited such that no more than *T*_*m*_ tests on susceptible and exposed individuals can be carried out at an instance. Let *H*(*x*) be the Heaviside step function that takes values *H*(*x*) = 0 for *x* < 0 and *H*(*x*) = 1 for *x* > 0,

$$\begin{array}{c} g=\kappa \frac{C}{C+I_{s}+I_{a}}H\left(T_{m}-T_{S}-T_{E}\right) \end{array}$$ (3)

fulfils the assumptions that we stated above. We assume the rest of the factors such as the ratio of false negative tests and likelihood of contact tracing are averaged into the fit parameter *κ*. In our implementation, we approximate the step function as *H*(*x*) ≈ 1/2 + (1/2) tanh(*x*). Because contact tracing is only possible through known cases, we expect the the probability *g* to increase with the ratio of identified cases to total number of infectious individuals and the ansatz (3) should be understood as the simplest expression that agrees with this intuition. While opting for model simplicity, we neglect beyond-linear-order terms, such as those proportional [*C*/(*C* + *I*_*s*_ + *I*_*a*_)]^3^ and [*C*/(*C* + *I*_*s*_ + *I*_*a*_)]^5^, and delays since taking the probability of an exposed case to be detected at time *t* to be a function of the number of cases and infectious individuals at time *t* ignores the latent time *γ*_*E*_ from exposure to become detectable.

Similar to *g*, the number of susceptible individuals to be tested per unit time *f* is also an unknown function of the social network and contact tracing procedures. Because in this case the individuals are not exposed to the pathogen, we assume that this rate is independent of the number of infectious individuals at present and take

$$\begin{array}{c} f=\left(\alpha SC/N\right)H\left(T_{m}-T_{S}-T_{E}\right)\;, \end{array}$$ (4)

as our second ansatz, where the term *αSC*/*N* is analogous to the *S* → *E* term of the SEIR model (Fig 1A). Here, we assume that the daily probability of a susceptible person to have a past contact with a known case wherein no infection have occured is proportional to the fit parameter *α* and is independent from the probability of being infected at the same time, i.e. the *S* → *E* transition. Once again, the step function *H*(*T*_*m*_ − *T*_*S*_ − *T*_*E*_) approximated as *H*(*x*) ≈ 1/2 + (1/2) tanh(*x*) models the capacity limit of contact tracing by setting *S* → *T*_*S*_ flux to 0, once the capacity limit *T*_*m*_ is reached. Finally, we ignore false positives, and thus let all individuals from the compartment *T*_*S*_ back to *S* after the turnover time 1/*γ*_*T*_.

As we illustrate through our results of the next section, the presence of *H*(*T*_*m*_ − *T*_*S*_ − *T*_*E*_) terms in (3) and (4) limits the total number of individuals in *T*_*S*_ and *T*_*E*_ compartments to *T*_*m*_ by substantially reducing *S* → *T*_*S*_ and *E* → *T*_*E*_ fluxes, as this limit is approached. This bound is not imposed upon the testing of the symptomatic individuals, i.e. the *I*_*s*_ → *T*_*s*_ term, since we assume it to be due to contact tracing. Finally, we assume that if an asymptomatic individual is detected via contact-tracing, this takes place before the individual becomes infectious, which is implied by the fact that those in *I*_*a*_ are not tested.

With the ansätze (3) and (4), the SEIRTC ODEs similar to (1) corresponding to the state transition diagram Fig 1B can be obtained by expressing the rates of change in compartment populations as the shown transition rates. Explicit form of these equations can be found in the S1 File (eqs. S1–S10). In our numerical results to follow, we simulate these using odeint function of scipy [17].

For model fitting and uncertainty quantification, we follow [18] and utilize weighted nonlinear least squares fit [19] for adjusting model parameters followed by a bootstrap method [20] for finding alternative sets of fit parameters. In a real-life scenario, testing constitutes the primary source of information as most countries publish the daily numbers of tests they conduct and those with a positive outcome (incidence). We make use of both of these measurements and minimize the cost function

$$\begin{array}{c} J=\sum_{n=0}^{N}\frac{1}{\tilde{T}}{\left(\tilde{T}\left[n\right]-T^{\prime }\left[n\right]\right)}^{2}+\sum_{n=1}^{N}\frac{1}{\tilde{C}}{\left(\tilde{C}\left[n\right]-C^{\prime }\left[n\right]\right)}^{2}\;, \end{array}$$ (5)

where [*n*] denotes the discrete time in days, ˜ indicates measurements coming from the surveillance data, and *T*′[*n*] = ∑_*i*=*S*,*E*,*I*_ *T*_*i*_[*n*](*γ*_*T*_ × day) and *C*′[*n*] = *C*[*n*] + *R*_*k*_[*n*] − *C*[*n* − 1] − *R*_*k*_[*n* − 1] are the number of tests carried out and new cases recorded in the model on day *n*, respectively. In the following, we take 1/*γ*_*T*_ = 1 day, which renders the factor *γ*_*T*_ × days = 1, i.e. each individual remains in the test compartment for 1 day. Note that because our definition of daily new cases *C*′[*n*] in the SEIRTC model depends on the total number of *C* + *R*_*k*_ of the previous day, the corresponding term in the cost function start from the day 1 rather than 0. The choice of weights ${\tilde{T}}^{-1}\left[n\right]$ and ${\tilde{C}}^{-1}\left[n\right]$ ensures that the optimization algorithm does not ignore the earlier stages in favor of the later days on which the case and test numbers are much higher. In order to reduce the number of fit parameters, we make the following simplifying assumptions. Whenever available, we take literature values for parameters or restrict them to the established estimated interval. While the number of exposed individuals on day 0 is varied, the initial populations of *T*_*S*_, *T*_*E*_, *I*_*a*_, *I*_*s*_, *T*_*I*_, *C* are adjusted through a fixed-point iteration that transfers individuals from *S* to these compartments while minimizing the error between the simulated dynamics of the first 10 days from exponential fits.*R*_*k*_(0) and *R*_*u*_(0) are both set to the number of cases registered until the first day. Although this is an arbitrary assumption for *R*_*u*_, it has no effect on the dynamics as long as it is much less than the total population, which is the case for our results to follow. With these assumptions, the set of fit parameters becomes *θ* = {*E*(0), *α*, *κ*, *T*_*m*_, *β*, *γ*_*s*_, *d*}. In our applications, we utilized least_squares method of scipy [17] to minimize (5) and find the best-fit parameters θ*. For bootstrapping, we generate synthetic data $\hat{T}\left[n\right]=\text{Pois}\left(T^{\prime }\left[n\right]\right),\hat{C}\left[n\right]=\text{Pois}\left(C^{\prime }\left[n\right]\right)$, where Pois(λ) indicates a random variable drawn from a Poisson distribution [21] with the mean and variance *λ*, and refit our model by taking the $\hat{T}\left[n\right],\hat{C}\left[n\right]$ as our new set of observations in (5). This procedure is illustrated in S2 Fig in S1 File of the supplement.

In the following for comparison, we also present fits by SEIR models for which we discard the test measurements from (5) and take the cost function

$$\begin{array}{c} J=\sum_{n=1}^{N}w_{C}\left[n\right]{\left(\tilde{C}\left[n\right]-C^{\prime }\left[n\right]\right)}^{2}\;, \end{array}$$ (6)

where *C*′[*n*] = *I*[*n*] + *R*[*n*] − *I*[*n* − 1] − *R*[*n* − 1] in the SEIR model. Similar to the SEIRTC model, we take *E*(0) and *β* as fit parameters and initiate simulations such that the dynamics of the first 10 days can be approximated by exponentials. In order to illustrate how the SEIR model fits the different stages of the time-interval considered for different weights, we consider two different choice of weights, namely *w*_*C*_[*n*] = 1 and $w_{C}\left[n\right]=1/\tilde{C}$.

In order to estimate the effective reproduction number (*R*_*t*_) from surveillance and model data we utilize the python implementation [22] of the [23]’s EpiEstim algorithm that is based on the Bayesian inference of *R*_*t*_ from a Gamma-distributed prior assuming Poisson-distributed transmissions. This method was also used by AGES [24] who performs the real-time epidemilogical monitoring of the ongoing COVID-19 situation in Austria.

## Results

**Fig 2:**
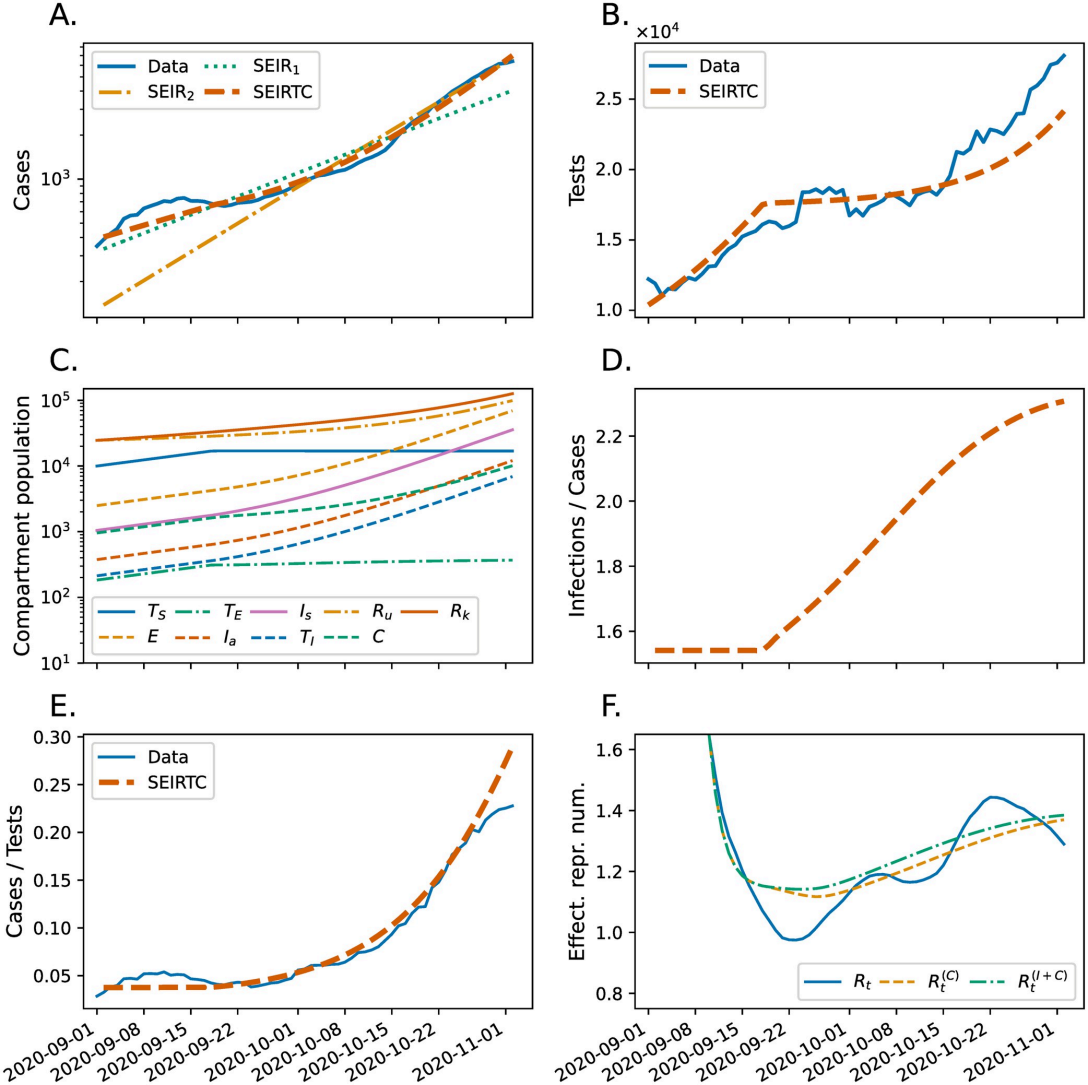
Case (**A**) and Test (**B**) data (7-day moving average) reported in Austria from September 1, 2020 to November 3, 2020 along with the fitting curves obtained from the SEIRTC model. Two SEIR model fits using different cost functions weights (see the main text) are also shown in **A** for comparison. **C**. Populations of the individual compartments (except *S*) of the SEIRTC model. **D**. The ratio of daily new infections to the cases in the SEIRTC model. **D**. The reported proportion of tests with a positive outcome and that in the SEIRTC model. **F**. Estimates *R*_*t*_, $R_{t}^{\left(C\right)}$, and $R_{t}^{\left(I+C\right)}$ of the effective reproduction number based on the reported case data, case numbers of the SEIRTC model, and combined case infection numbers of, respectively.

We consider the second wave of COVID-19 in Austria from September 1 to November 3, 2020, on which day the country went into its second lockdown in order to protect its healthcare system from an otherwise-inevitable overload. Fig 2A and 2B shows the 7-day moving averages of the numbers of confirmed cases and performed tests during this period, respectively (retrived from [14]). Fits to these data by the SEIRTC model with parameters in Table 1 are also shown in Fig 2A and 2B, which we obtained by minimizing (5). For comparison in Fig 2A, we also show fits by SEIR models with initial conditions and model parameters as listed in Table 2. The different choice of weights in (6) results in *SEIR* models, with different set of model parameters and initial conditions, which we refer to as SEIR_1_ and SEIR_2_. As shown in Fig 2A, when unit weights are chosen, the SEIR_2_ model underestimates the initial case numbers whereas when the weights are inversely proportional to the daily number of confirmed cases, the later case numbers are underestimated by the SEIR_1_ model. In contrast, the fit by the SEIRTC model captures both episodes. This is further illustrated by the scatter plots of model predictions against the observations in S1 Fig in S1 File where the largest deviation between the two are also marked for each model. Quantitatively, the largest percentage relative error between the case numbers and their model predictions, i.e. $100\times {\text{max}}_{n}\left(\tilde{C}\left[n\right]-C^{\prime }\left[n\right]\right)/C^{\prime }\left[n\right]$, are 39.3% and 68.8% for SEIR_1_ and SEIR_2_ models, respectively, whereas it is 25.0% for the SEIRTC model. In addition, we also carried out a reduced-*χ*^2^ “goodness of fit” test taking our model predictions as means of Poisson distributions, see the S1 File for details. $\chi_{\nu }^{2}=\chi^{2}/\nu$ where *ν* is the number of degrees of freedom is $\chi_{\nu }^{2}=223.1$ and $\chi_{\nu }^{2}=207.1$ for SEIR_1_ and SEIR_2_ models, respectively, whereas $\chi_{\nu }^{2}=86.7$ for the SEIRTC model. The lower $\chi_{\nu }^{2}$ of the SEIRTC model further demonstrates that it is a better fit to the data considered [25]. In addition to the goodness of fit test, we also performed a Poisson bootstrap analysis to reveal parameter correlations of the SEIRTC model. Although some of the model parameters show correlations (see S3 Fig in S1 File) our main results are robust to these parameter variations.

**Table 1:** The model parameter values which are used in the fit shown in Fig 2.

Symbol	Value [Ref.] / [Fit]	Symbol	Value [Ref.] / [Fit]
N	8894380 [26]	*β*	0.5993 / person / day [Fit]
*α*	10.84 / person / day [Fit]	*κ*	0.7546 [Fit]
p	0.2 [27]	d	0.5446 [Fit]
1/*γ*_*E*_	4 days [28]	1/*γ*_*T*_	1 day [29]
1/*γ*_*I*_	5 days [28]	1/*γ*_*s*_	2.594 days [Fit]
*T*_*m*_	17 252 [Fit]	*ρ*	0.35 [27]

**Table 2:** Cost function weights and parameters for SEIR models with fit curves shown in Fig 2.

Model	*w*_*C*_[*n*]	1/*γ*_*E*_	*γ*_*I*_	N	E(0)	I(0)	R(0)	*β*
SEIR_1_	$1/\tilde{C}\left[n\right]$	4 days [28]	5 days [28]	8894380 [26]	1306	1353	5390	0.282
SEIR_2_	1	4 days [28]	5 days [28]	8894380 [26]	535	506	1518	0.332

As shown in Fig 2B, the SEIRTC model also captures the reported test numbers with a visible change in its trend during the days following September 15. This coincides with the exhaustion of the contact tracing capacity as reflected by the plateaus of *T*_*S*_ and *T*_*E*_ in Fig 2C. Consequently, the ratio *I*′[*n*]/*C*′[*n*] (Fig 2D), where *I*′[*n*] = ∑_*i*_ *I*_*i*_[*n*] + ∑_*i*_ *R*_*i*_[*n*] + *C*[*n*] − ∑_*i*_ *I*_*i*_[*n* − 1] + ∑_*i*_ *R*_*i*_[*n* − 1] + *C*[*n* − 1], of the daily new infections to those that are detected starts growing, yielding more and more infections that remain undetected. An observable consequence of this is the increase of the proportion of the tests with a positive outcome as shown in Fig 2E where we plotted this quantity using the data reported and those obtained from the SEIRTC model.

In Fig 2F, we show the estimates of the effective reproduction number *R*_*t*_ calculated using the case data shown in Fig 2A and those computed from the SEIRTC model’s daily case (orange, dashed) and infections (green, dotted-dashed). In these calculations, we chose a smoothing window of 7 days and used a serial interval obtained by discretizing a Gamma distribution with a mean 4.46 and a standard deviation 2.63 days as estimated by AGES [24].

**Fig 3:**
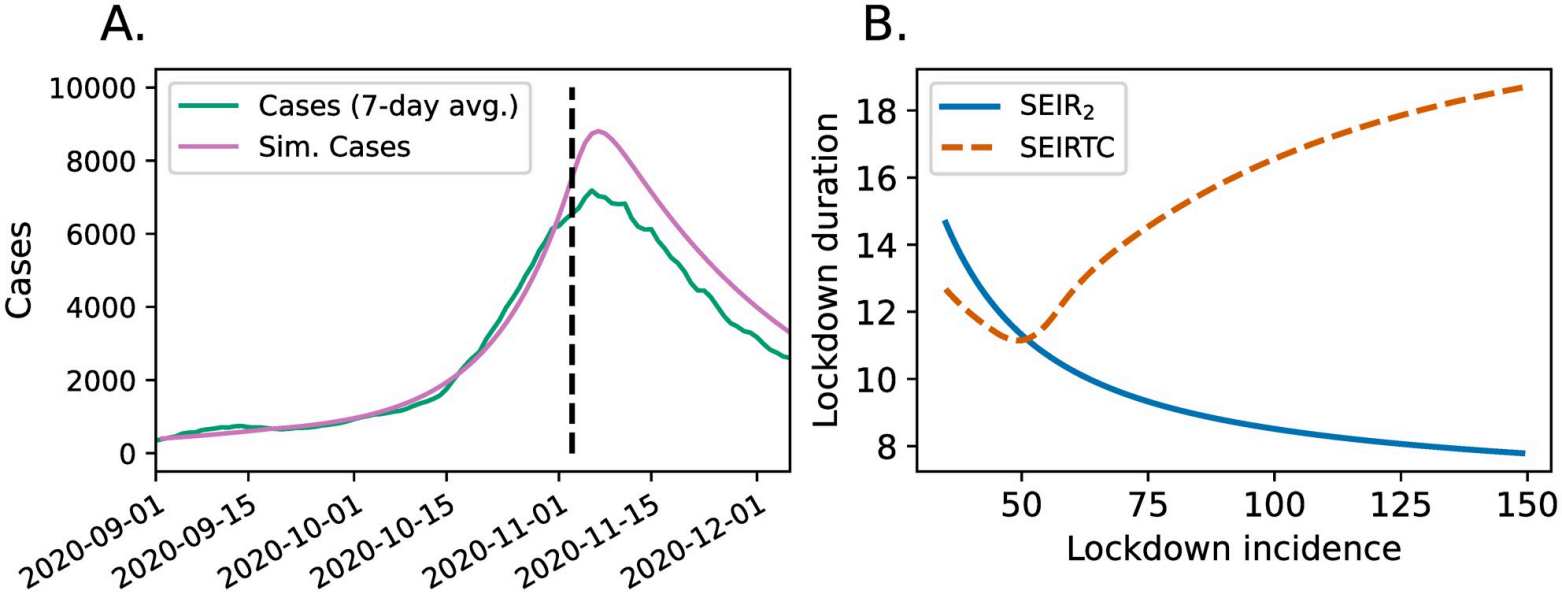
**A**. Case data reported in Austria from September 1, 2020 to December 6, 2020 along with its prediction by SEIRTC model wherein the lockdown is modeled by a reduction of *β* to 45% of its value on November 3 which is indicated by the dashed line segment. **B**. Number of days in “lockdown” necessary for reducing the 7-day incidence (per 100,000) by 10 as a function of the incidence at which the lockdown begins. Lockdown is modeled by a drop of the transmission parameter *β* to half of its value.

About a week after the beginning of lockdown in Austria on November 3 2020, the case numbers began to decrease as shown in Fig 3A. We observed that this trend can be qualitatively captured by the SEIRTC model if the transmission parameter *β* is reduced to 45% of its value on the same day as shown in Fig 3A. Taking this as a crude model of a lockdown, we investigated hypothetical scenarios during which the transmission parameter *β* is reduced to 0.45*β* beginning at different times. Specifically, we started SEIR_2_ and SEIRTC models with their initial conditions and model parameters same as those that we used in Fig 2, initiated lockdowns when the 7-day incidence (per 100,000 people) reached a certain value, and measured the lockdown duration necessary for reducing the incidence by 10. The results of these simulations are shown in Fig 3.

## Discussion

Fig 2A and 2B demonstrates that the SEIRTC model with parameters listed in Table 1 and the initial population shown in Fig 2C is able to capture the accelerating spread of COVID-19 observed in Austria in Fall 2020. We note that some of these fit parameters are correlated, hence, it is possible to find different set of parameters that result in comparable fitting errors, see S2 and S3 Figs in S1 File. Most of the correlations seen in S3 Fig in S1 File are easy to rationalize. For example, the negative correlation of *E*(0) and *α* tells us that if the number of exposed on day zero is increased, then the probability of testing susceptibles has to decrease to free capacity for the exposed. A similar argument can be made for the negative correlation of *κ* and *α*, which are proportional to the rate of testing exposed and susceptibles, respectively. For predicting the future of an ongoing outbreak, quantification of uncertainties due to such parameter correleations is crucial and, thus, should be carefully taken into consideration [18]. Because near-real-time prediction is not our goal here, we focus on the qualitative aspects of our findings that are robust to parameter uncertainties.

While Fig 2A illustrates how an autonomous SEIR model cannot capture an accelerating epidemic, it also suggest how a nonautonomous SEIR model with a time-varying transmission parameter *β*(*t*) could have indeed describe the observed case numbers. One could then have interpreted the accelerating spread as being due to the seasonal effects such as people spending more time indoors hence increasing chance of transmission. Another—somewhat trivial—modification to the SEIR model could have been addition of infectious individuals to the model by hand as a proxy for people bringing the virus from outside the country through travel as suggested by the Austrian then-Chancellor Sebastian Kurz who claimed that the sudden increase of the COVID-19 cases during Fall 2020 was largely due to the Austrians of foreign origin who brought the virus back from their their countries of origin [30]. It is conceivable that both of these factors have played some role in the sudden spread of COVID-19 in Austria in Fall 2020 however they as such do not explain the coinciding increase in the test-positive rate. As we point out in this study capacity limits in mitigation have played a key role in the epidemic acceleration and offer an explanation for the observed events including the change in positive rate.

The methods for estimating the effective reproduction number are usually believed to be robust against incomplete observations under the assumption that an approximately same fraction of infections are recorded on consecutive days [31]. This is also reflected in our initial estimates of the effective reproduction number, since when the ratio of the number of infections to that of confirmed cases is constant as in the initial phase of Fig 2D, $R_{t}^{\left(C\right)}$ and $R_{t}^{\left(I+C\right)}$ coincide in Fig 2F. This, however, breaks down as soon as the contract tracing limit is reached; after this point, the effective reproduction number based only on the case data systematically underestimates the one that is based on the actual number of infections. Although the difference between $R_{t}^{\left(C\right)}$ and $R_{t}^{\left(I+C\right)}$ appear small in Fig 2F, it should be recalled that this difference translates into the number of infections exponentially, thus, has a dramatic real-life consequences such as expected number of hospital admissions. Note that the initial overshoot of the effective reproduction number in Fig 2F is due to our omission of case data prior 2020–09-01, which results in an overestimate of the effective reproduction number.

As one should expect, the increase of undetected cases in Fig 2D coincide with that of the ratio of positive tests in Fig 2E, which is observable during an epidemic. While this information could, and probably should, be incorporated into statistical methods for estimating *R*_*t*_, we believe that an increasing positive test ratio is a sufficient reason for a dramatic intervention such as a lockdown, which is essentially inevitable once the contact tracing capacity is exhausted.

During the second wave of covid-19 in Austria, the policy makers insisted that a lockdown would be the last option in the country’s pandemic response [32]. Decreasing lockdown durations for the SEIR model as shown in Fig 3B might indeed suggest this as a reasonable compromise to minimize the number of days during which the economic and social activities are halted. Note, however, that this behavior changes dramatically in the SEIRTC model since the uncontrolled spread following the breakdown of contact tracing makes it progressively harder to reduce the case numbers. This is why we believe that a steadily increasing ratio of positive tests necessitates a lockdown. At that point, early action not only saves lives but also shortens the lockdown duration necessary to regain control.

## Supporting information

S1 File Explicit form of the SEIRTC model equations, further comparisons of fits by SEIR and SEIRTC models, and parameter uncertainty analysis via Poisson-bootstrap method are presented in the supplementary material. (PDF)
