# Supplementary material for: An autonomous compartmental model for accelerating epidemics
Source: PLoS One. 2022 Jul 18;17(7):e0269975. doi: 10.1371/journal.pone.0269975 (PMC9292088; doi:10.1371/journal.pone.0269975)
Supplement: S1 File — (PDF) [file pone.0269975.s001.pdf]

Supplementary Material for "An autonomous compartmental model for accelerating epidemics"

Nazmi Burak Budanur*[1,2] and Björn Hof [2]
[1]Max Planck Institute for the Physics of Complex Systems (MPIPKS)
Nöthnitzer Straße 38, 01187 Dresden, Germany
[2]Institute of Science and Technology Austria (IST Austria)
Am Campus 1, 3400 Klosterneuburg, Austria

July 6, 2022

SEIRTC equations

The set of ODEs that correspond to the SEIRTC model shown in Figure 1B read

$$\begin{aligned}
\dot{S} &= -f + T_S\gamma_T - \frac{S\beta\left(\rho I_a + I_s\right)}{N} && \text{(S1)}\\
\dot{T_S} &= f - T_S\gamma_T && \text{(S2)}\\
\dot{E} &= -gE - E\gamma_E p(1-g) - E\gamma_E\left(1-p\right)\left(1-g\right) + \frac{S\beta\left(\rho I_a + I_s\right)}{N} && \text{(S3)}\\
\dot{T_E} &= gE - T_E\gamma_T && \text{(S4)}\\
\dot{I_a} &= E\gamma_E p(1-g) - I_a\gamma_I && \text{(S5)}\\
\dot{I_s} &= E\gamma_E\left(1-p\right)\left(1-g\right) - I_s d\gamma_s - I_s\gamma_I\left(1-d\right) && \text{(S6)}\\
\dot{T_I} &= I_s d\gamma_s - T_I\gamma_T && \text{(S7)}\\
\dot{R_u} &= I_a\gamma_I + I_s\gamma_I\left(1-d\right) && \text{(S8)}\\
\dot{C} &= -\gamma_C C + T_E\gamma_T + T_I\gamma_T && \text{(S9)}\\
\dot{R_k} &= \gamma_C C && \text{(S10)}
\end{aligned}$$

where γ_C, g, and f are given by (2), (3), and (4), respectively.

Comparison of fits

Figure S1 presents a visual comparison of the fits by *SEIR* and *SEIRTC* models where the recorded cases are plotted against the model predictions. The red arrows indicate the maxima of relative prediction errors.

*nbudanur@pks.mpg.de

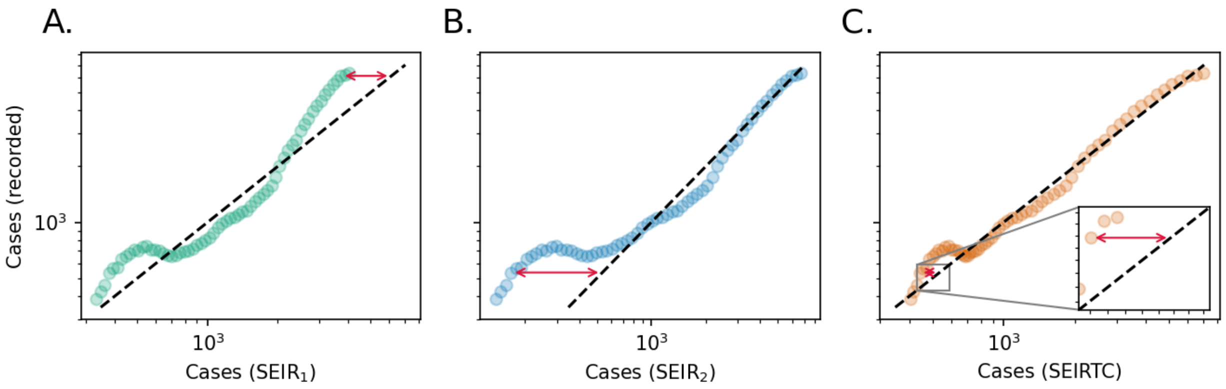

Figure S1: **Comparison of fits.** Scatter plots show the recorded case incidences against the **A.** SEIR$_1$, **B.** SEIR$_2$, and **C.** SEIRTC model predictions. Red arrows indicate the locations of largest relative mismatch between the recorded incidence and model predictions which are equal to 39.3% (**A**), 68.8% (**B**), and 25.0% (**C**).

As a "goodness of fit" test [1], we compute the reduced chi-squared $\chi^2_\nu = \chi^2/\nu$ where ν is the number of degrees of freedom and

$$\chi^2 = \sum_i \frac{\tilde{X}_i - X_i}{\sigma_i^2} \,. \tag{S11}$$

Here, the sum is over the observations, $\tilde{X}_i$ is the observed value, such as the case numbers on the i-th day, X_i is its model prediction and σ_i^2 is its variance. Since our models only predicts the means and we have one observation per day, we do not have a straightforward way of estimating the variance of the case and test numbers, which are the observables. We, thus, resort to the Poisson assumption, i.e. variance equal to mean, and compute (S11) assuming $\sigma_i^2 = X_i$. Finally, we take the number of degrees of freedom to be $\nu = n_{obs} - n_{pars}$, where n_{obs} and n_{pars} are the number of observations and fit parameters, respectively. Since we fit the SEIR models only to the case data, only the case numbers go into the calculation of (S11), whereas for SEIRTC we take both case and test data into account. Consequently, the number of observations is nearly twice as many in the latter case.

Parameter uncertainties

Bootstrapping method for quantifying parameter uncertainty consists of generating synthetic data sets from the best-fit model by adding noise with a presumed error structure and refitting the model to the newly-generated data [2]. As a result, one obtains different sets of parameters which performs similarly in the training data, which can then be further analyzed to reveal the parameter correlations. We illustrate this procedure in Figure S2 where synthetic datasets generated randomly under the assumption of Poisson-distributed errors are shown along with the predictions from the best-fit model (Figure S2A,B). For clarity of illustration here we only show the first 20 days but the synthetic data were generated for the entire time

interval we consider. After generating a synthetic data, we fit the model parameters to this newly generated data set, and repeated this procedure for 100 realizations. Resulting model predictions differ slightly as illustrated in Figure S2C,D.

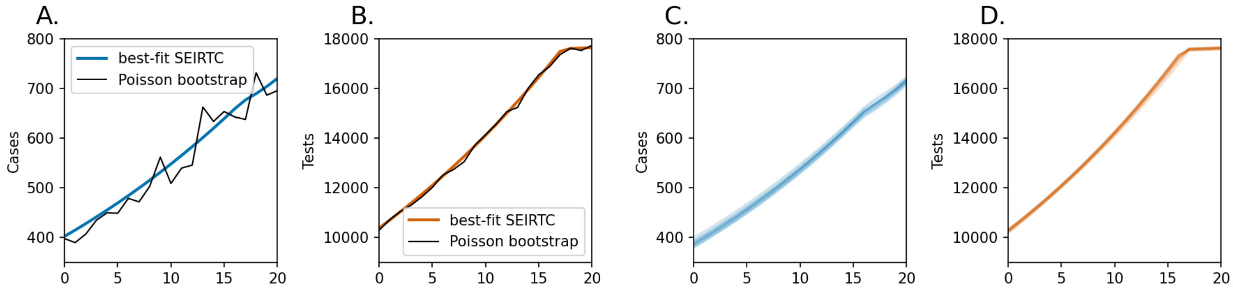

Figure S2: **Parametric bootstrap method.** Case (**A**) and tests (**B**) data (first 20 days) predicted by the best-fit SEIRTC model along with synthetic data generated by assuming Poisson-distributed uncertainties around the means predicted by the SEIRTC model. Case (**C**) and test (**D**) data (first 20 days) predicted by the SEIRTC models with 10 different sets of parameters that are obtained by re-fitting the model to synthetic data such as those illustrated in **A** and **B**.

In order to reveal parameter correlations of the SEIRTC model, we plot the best-fit parameters of bootstrap realizations against one another in Figure S3. Here all-but-one of the 100 bootstrap realizations is shown, where we ruled one of the realizations an "outlier" for having best-fit parameters more than three standard deviations far from the means.

References

[1] R. J. Barlow. *Statistics: A Guide to the Use of Statistical Methods in the Physical Sciences*. Manchester Physics Series. Wiley, 1993. ISBN 9780471922957.

[2] G. Chowell. Fitting dynamic models to epidemic outbreaks with quantified uncertainty: A primer for parameter uncertainty, identifiability, and forecasts. *Infectious Disease Modelling*, 2(3):379–398, aug 2017. doi: 10.1016/j.idm.2017.08.001. URL https://doi.org/10.1016%2Fj.idm.2017.08.001.

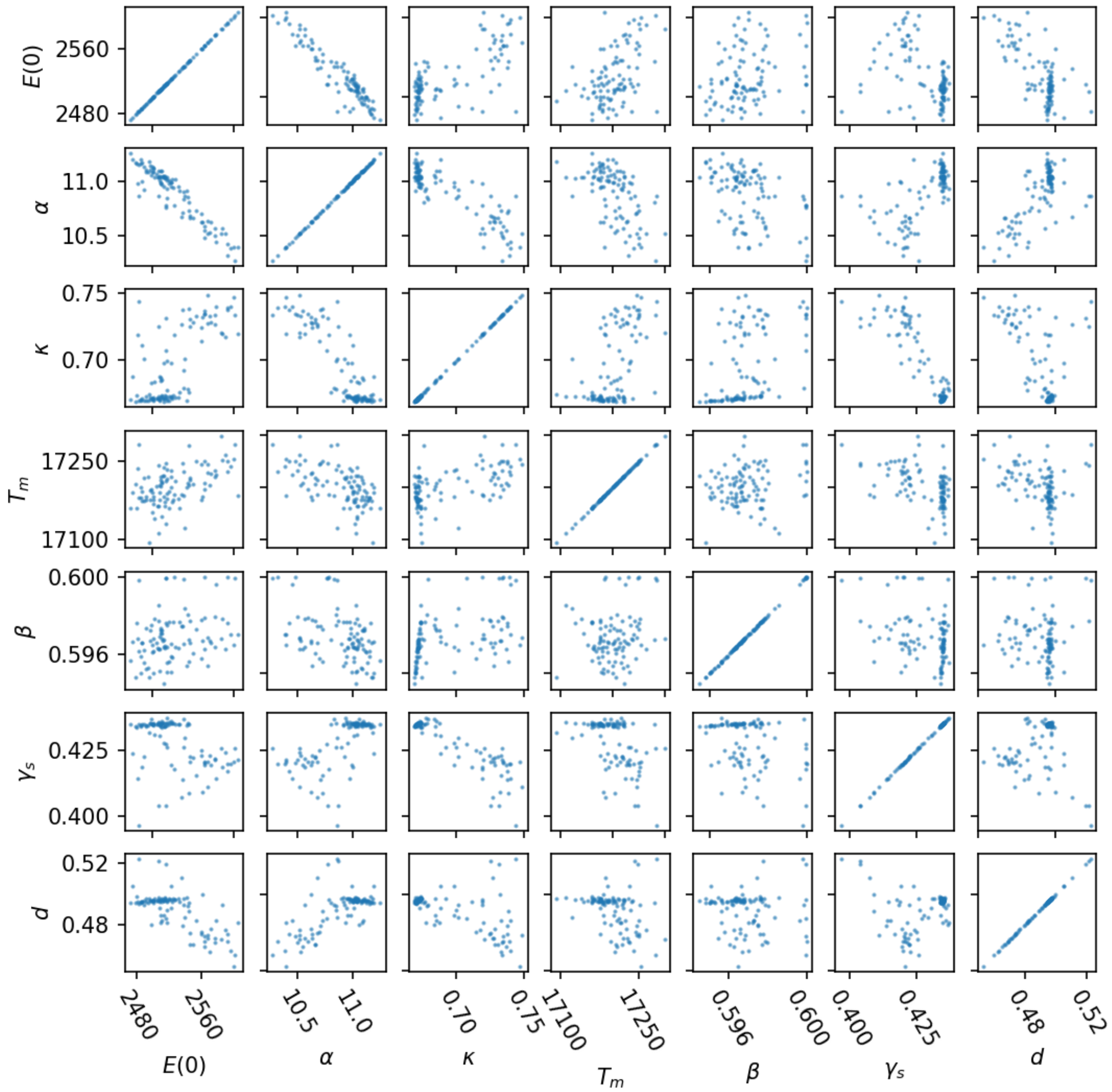

Figure S3: **Scatters of SEIRTC model parameters.** Each panel shows the scatter of a pair of parameters obtained by re-fitting the SEIRTC model to synthetic datasets obtained by the Poisson bootstrap method.
